# Supplementary material for: Acute pain and analgesic requirement after vaginal childbirth with and without neuraxial labor analgesia–Retrospective cohort study
Source: PLoS One. 2023 Apr 18;18(4):e0284106. doi: 10.1371/journal.pone.0284106 (PMC10112800; doi:10.1371/journal.pone.0284106)
Supplement: Attachment — Submitted filename: Response to Reviewers.docx [file pone.0284106.s001.docx]

**Response to Reviewers**

Reviewer #1:

- This manuscript presents a retrospective cohort study to investigate the association between the use of neuroaxial labor analgesia and outcomes related to postpartum. Overall, the manuscript is well written.
  - Thank you very much. We really appreciate your positive feedback.
- However, the major issue is that the research hypothesis is not clear. The literatures about the clinical outcomes associated with neuraxial analgesia should be mentioned.
  - We added our hypotheses at the end of Introduction section.
  - We added another literature that performed a meta-analysis on the analgesic effect of labor epidural. Unfortunately, the literature on the association between the neuraxial labor analgesia and postpartum pain is limited, but the relevant references are cited in the Introduction section.
- Minor issues include (1) Line 312, the results from this retrospective cohort study based on chart review cannot draw any conclusion
  - We completely agree and made alterations in our manuscript to clarify that we are not trying to draw any conclusion based on this retrospective study.
- (2) Line 265, the difference between those who delivered with and without neuraxial analgesia is equivalent to only 0.4 point on 10-point NRS scores. Comparing to severe maternal morbidity, how these research findings help clinicians? What contribution or importance do these findings have to the clinical practice?
  - We agree that the difference is extremely small and not clinically significant even though it is statistically significant. This result is unlikely to change our clinical practice. Based on the comments from both Reviewers, we decided to alter our conclusion to ensure that our results will not mislead the audience to a false alarm that epidural will increase postpartum pain.
- Other questions: in Table 1 several baseline characteristics were significant differences between two groups. This comparison feels weird. How to compare the outcome based on two different participants?
  - We recognized that several baseline characteristics (in Table 1) and obstetrical outcomes (in Table 2) were different between the two groups. Therefore we performed logistic regression analysis to adjust for these potential confounders.
- Besides, why the authors use median (IQR) instead of mean (SD) in comparison of continuous data?
  - The histograms for each parameter revealed skewed distribution of the data. Therefore, we decided to use non-parametric tests (Mann-Whitney U test) that provide us median with IQR, instead of using parametric tests (t test) that provide mean (SD).

Reviewer #2: PONE-D-22-14620

- The authors report an observational study comparing pain the first five days after vaginal delivery, between women receiving (39% of cohort) and not receiving (61% of cohort) epidural labor analgesia (ELA). The authors report that those receiving ELA experience worse pain in the postpartum period. The major concerns about this paper include the way that postpartum pain was measured/calculated, and the imbalance between ELA use and non-use in the cohort, both of which can significantly increase the risk for Type 1 error. The other major concern centers around conclusions based on statistical significance, without balancing clinical relevance / clinical significance.
  - We certainly recognize the two major concerns you pointed out as the limitation of this retrospective study. We agree that there exists a significant risk of Type 1 error, and therefore made alterations to our Discussion and Conclusion sections to ensure that our results will not mislead the audience to a false alarm that epidural will increase postpartum pain. Thank you very much for your precious advice.
- Major concerns
  - We truly appreciate your important feedbacks and did our best to reflect all aspects of your comments to our updated manuscript. Thank you very much.
- 1. The postpartum pain AUC that was calculated was not corrected for assessment times. It is not clear whether pain assessments were taken at regular intervals for each patient, missingness of data, irregularity of assessments, etc. as often occurs in normal clinical care. The variability of assessments means that a standard AUC for all assessments can OVERESTIMATE the measurement of pain (higher AUC) among patients who have more pain assessments (bias: patients with more pain have more assessments due to nurses presumably intervening to help control that pain) compared to those who do not complain of pain / do not have it assessed as often (subsequent UNDER ESTIMATION of pain assessments). In turn, the risk for Type 1 error is systematically elevated and makes the conclusions of the paper more than likely to be false.

2. Even considering the measurement problems, the differences found for peak NRS (median 4 [IQR 3–5] vs. 3 [2–4], p< 0.001) are not clinically significant, even though they meet definition of statistical significance. Said differently, the difference between median pains core reporting 3 vs 4 is really not clinically relevant.

- We added explanation to clarify that pain assessments were not performed at regular time intervals and therefore there is a possibility that pain scores were either overestimated or underestimated as you suggested.
- The risk for Type 1 error is unfortunately inherent to this study design. We altered our Discussion and Conclusion to clarify that the small difference we found statistically significant is unlikely to be clinically meaningful.
- 3. The cohort has significantly lower proportion of people receiving ELA than not receiving ELA. Given the imbalance, it is likely that those who are requesting ELA are different (in both measurable and immeasurable ways) from the rest of the cohort. For example, induction of labor (IOL) is more painful and associated with increased ELA utilization, and IOL is also associated with worse postpartum pain (i.e., complete confounding). In turn, the risk for Type 1 error is systematically elevated and makes the conclusions of the paper more than likely to be false.
  - In Table 1 and 2 we recognized significant differences in background characteristics between the two groups. Therefore we decided to perform logistic regression in Table 4-6 to adjust for potential confounders. It is true, however, that other unmeasured factors (such as each patient’s pain tolerance) confounded the results. We made alterations to Discussion section to emphasize these limitations.
- 4. Similarly, the secondary findings of increased medication use (diclofenac, acetaminophen, etc) among the ELA cohort may not imply causality, but rather simply indicate a cohort of patients who are more likely to use medications for any pain. The fact that the differences between groups for peak NRS and AUC were not clinically significant between the groups (despite statistical significance)
  - We agree with your hypothesis and updated the manuscript to emphasize that we should not determine any causality based on these results.
- 5. The conclusions are stated rather boldly that ELA is associated with worse postpartum pain. To uninformed audience this could be very alarming and cause people to draw extreme conclusions about ELA leading to worse postpartum outcomes.
  - To mislead our audience to such false alarm and wrong conclusion is not our intention. We really appreciate your feedback and changed our conclusion to prevent any misinterpretation.
- 6. The Abstract conclusions suddenly bring up hemorrhoids but there is no data in the methods/results that support that idea.
  - The conclusion was drawn based on the results shown in Table 2 and 6 and accompanied by discussion in line 293 – 307. However, we updated the manuscript to ensure that we do not imply any causality.
- 7. Most commonly, pain after ELA is localized back pain which is typically mild and self-limited, not debilitating or clinically disabling for patients. What is to say that the 3 vs 4 NRS difference is not stemming from very mild back pain?
  - That is a great point and we added a discussion on localized back pain in line 250-254. We agree that the pain is not debilitating or clinically disabling, and re-iterated that it is unlikely to be clinically meaningful throughout our Discussion section.
