# Supplementary material for: Acute pain and analgesic requirement after vaginal childbirth with and without neuraxial labor analgesia–Retrospective cohort study
Source: PLoS One. 2023 Apr 18;18(4):e0284106. doi: 10.1371/journal.pone.0284106 (PMC10112800; doi:10.1371/journal.pone.0284106)
Supplement: Attachment — Submitted filename: Response to Reviewers 031523.docx [file pone.0284106.s002.docx]

**Response to Reviewers**

Journal Requirements:

- Please review your reference list to ensure that it is complete and correct. If you have cited papers that have been retracted, please include the rationale for doing so in the manuscript text, or remove these references and replace them with relevant current references. Any changes to the reference list should be mentioned in the rebuttal letter that accompanies your revised manuscript. If you need to cite a retracted article, indicate the article’s retracted status in the References list and also include a citation and full reference for the retraction notice.
  - We reviewed our reference list and it seems complete and correct. We added one reference (#9 – please see below) and made sure that we are using Vancouver style. If there is any reference that needs to be updated or replaced, please kindly let us know.

Additional Editor Comments:

- I suggest that “neonatal birth weight” should be included as a confounder for NRS-AUC5days and peak NRS ≥4. Because neonatal birth weight makes an important influence on NRS pain score. Besides, there is a significant difference in “neonatal birth weight” between the two groups in Table 2.
  - Thank you for this very important suggestion. We included neonatal birth weight and updated Tables 4 and 5.
- Dose gestational age belong to maternal characteristic or obstetrical outcome? Please keep consistent classification through all tables.
  - We moved “gestational age” from Obstetrical outcomes to Maternal characteristics in Tables 4-6.
- Please provide the crude OR and P value in Table 4-Table 6.
  - Added crude ORs and p-values in Tables 4-6.
- Please keep unified description for the confounders in all tables. For example, there is “induction of labor” in Table 2 but “induction” in Table 4-Table 6.
  - We updated Table 1, 4-6 so the descriptions for the confounders are consistent.
- 4. Have the authors made all data underlying the findings in their manuscript fully available? Reviewer #3: Yes, Reviewer #4: No
  - We uploaded our dataset to Harvard Dataverse (https://dataverse.harvard.edu).

Reviewer #3:

- Overall, the revisions have resulted in a much better manuscript, however, it could still be written more succinctly; moreover, would recommend the omission of additional words that are ambiguous (“relatively”, “better”, etc.; see specific comments, but review paper), and revision of sentences that end in prepositions (“with”, “without”).
  - We omitted ambiguous words as suggested, and revised the sentences that were ending in prepositions.
- Can the authors provide a reference for the AUC pain equation used?
  - We referred to articles #5 (Kingsnorth), #6 (Smith) and #7 (Matthews). Our equitation was established based on the equations used in #5 and #6, with an addition of maxNRS to the denominator to standardize the scale.
- There are some papers/websites that convert blood volume to weight (which indicate 1cc = 1.06 gms; thus, the conversion in the paper is acceptable), but should be referenced; this is to distinguish the calculation from those papers that evaluate cc of clot to gms (which can be different).
  - We added another reference (#9: ACOG Committee Opinion No.794) which suggests that 1 gram weight blood loss should be converted to 1 milliliter blood loss volume and vice versa.
- An improvement in the paper could be achieved from moving away from short, separated sentences to more cohesive sentences.
  - We made several alterations to the original manuscript to minimize short, separated sentences.
- In terms of postpartum hemorrhoids, there appear to be risk factors that should be discussed, that were identified in the results: “older, with higher pre-delivery BMI and advanced gestational age, in higher proportion primiparous, and with significantly higher rates of hypertensive disorder of pregnancy and pre-existing hemorrhoids.”
  - We performed logistic regression analysis to adjust for these risk factors (please see Table 6). We edited the discussion (ln 329) to emphasize this.
- Pg 38, ln 108. Would suggest omitting “relatively”. What is meant by this word, and does it’s omission change the sentiment?
- Pg 38, ln 108. As an example of extra words that could be omitted, this hypothesis statement could be simplified to be “We hypothesized that women receiving neuraxial labor analgesia would have lower pain tolerance, and higher postpartum pain scores and analgesic requirements”
  - We simplified our hypothesis statement as suggested. Thank you for your suggestion.
- Pg 40, ln 138. Either remove PRN, or add (pro re nata; as needed)
  - Added “pro re nata” as suggested. Thank you.
- Pg 40, ln 144. Remove “better”.
  - Removed “better”.
- Pg 40, ln 147. Succinct: “as area under the curve (AUC) of numeric rating scale (NRS) for pain, using the anchors of 0 to 10 (no pain, worst pain imaginable, respectively).
  - Simplified the sentence as suggested. Thank you.
- Pg 46, ln 284. Please alter to: “Although the use of labor epidural analgesia does not increase the risk of long term lower back pain…”
  - Altered the sentence as suggested. Thank you very much.

Reviewer #4:

- Authors basically have addressed reviewers’ comments and concern. I think it is meaningful to publish this manuscript. I add the following suggestions for consideration.
  - We really appreciate your comments. Thank you very much.
- Abstract
  1)In Line 43, rephrase the sentence because current statement is strong to show the causal relationship between the use of neuraxial labor analgesia and postpartum pain. The statement could be “ it is unknown if the use of neuraxial labor analgesia is related to reported postpartum pain and analgesia consumption.
  - (We are assuming that Line 43 refers to the first sentence of Conclusion in Abstract.) We rephrased the conclusion to avoid overinterpretation of our results. Also added our recommendation at the end of the Conclusion.
- 1)Authors could provide research or clinical recommendations in the Conclusion part.
  - We added our recommendation for clinical practice at the end of the Conclusion (“should not influence women’s choice to receive labor analgesia”).
- 2.Materials and Methods
  1)One concern I have is about days of hospitalization after delivery. Certainly, It varies among different countries. In our country, women with regular vaginal delivery, are discharged within 2 days after discharge. I think it is even shorter in western countries. I think authors need to mention this in the discussion part.
  - We discussed this as one of the limitations of our study.
- 2)About the name of research variable, I suggest authors to use “level of pain” or “reported pain” instead of “pain burn” because it is measured using NRS only.
  - Changed the words “pain burden” to “level of pain”, “reported pain” etc. as suggested. Thank you.
- 3.Discussion
  2)From line 233 to line 242, in beginning of this finding should be clearly stated as “the use of neuraxial labor analgesia was associated with significantly higher pain scores during first five days postpartum”, and explain possible reasons and compare your finding with other studies. After this, you state this finding should be interpreted with cautions because of the following reasons.......
  - We used the word “slightly”, instead of “significantly”, understanding that this small statistically-significant difference is unlikely to be clinically significant.
- 2)From your main findings, authors need to make some recommendations for further research and/or clinical practice.
  - We added our recommendation for clinical practice in the Discussion and at the end of the Conclusion (“should not influence women’s choice to receive labor analgesia”).
